# Something Must Be Wrong with the Implementation of Cancer-pain Treatment Guidelines. A Lesson from Referrals to a Pain Clinic

**DOI:** 10.5041/RMMJ.10369

**Published:** 2019-07-18

**Authors:** Gil Samuelly-Leichtag, Tsiki Adler, Elon Eisenberg

**Affiliations:** 1 The Ruth & Bruce Rappaport Faculty of Medicine, Technion–Israel Institute of Technology, Haifa, Israel; 2 Pain Research Unit, Institute of Pain Medicine, Rambam Health Care Campus, Haifa, Israel

**Keywords:** Cancer-related pain, pain specialist referral, treatment, WHO guidelines adherence

## Abstract

**Objective:**

The World Health Organization’s (WHO) guidelines for cancer pain management were intentionally made simple in order to be widely implemented by all physicians treating cancer patients. Referrals to pain specialists are advised if pain does not improve within a short time. The present study examined whether or not a reasonable use of the WHO guideline was made by non-pain specialists prior to referral of patients with cancer-related pain to a pain clinic.

**Methods:**

Cancer patients referred to a pain specialist completed several questionnaires including demographics, medical history, and cancer-related pain; the short-form McGill Pain Questionnaire (SF-MPQ); and the Short Form Health Survey SF-12. Data from referral letters and medical records were obtained. Treatments recommended by pain specialists were recorded and categorized as “unjustified” if they were within the WHO ladder framework, or “justified” if they included additional treatments.

**Results:**

Seventy-three patients (44 women, 29 men) aged 55 years (range, 25–85) participated in the study. Their pain lasted for a mean of 6 (1–192) months. Mean pain intensity scores on a 0–10 numerical rating scale were 7 (2–10) at rest and 8 (3–10) upon movement. Most patients complied with their referring physician’s recommendations and consumed opioids. Adverse events were frequent. No significant correlation was found between the WHO analgesic medication step used and mean pain levels reported. There were 63 patient referrals (85%) categorized as “unjustified,” whereas only 11 patients (15%) required “justified” interventions.

**Conclusions:**

These findings imply that analgesic treatment within the WHO framework was not reasonably utilized by non-pain specialists before referring patients to pain clinics.

## INTRODUCTION

The treatment of cancer-related pain is a primary goal for healthcare professionals. Pain affects two-thirds of patients with advanced cancer, reaching intensities of moderate-to-severe in more than half of them.^1^ Pain therefore has a significant impact on cancer patients and can lead to depression, anxiety, and disability.^2^,^3^ Not surprisingly, patients with cancer report pain as one of the most feared symptoms of cancer and its treatments, and over one-third of patients label it as distressing or intolerable.^4^ The magnitude and severity of the problem was the main reason for the development of the World Health Organization’s (WHO) guideline for cancer pain management.^5^

**Figure 1:**
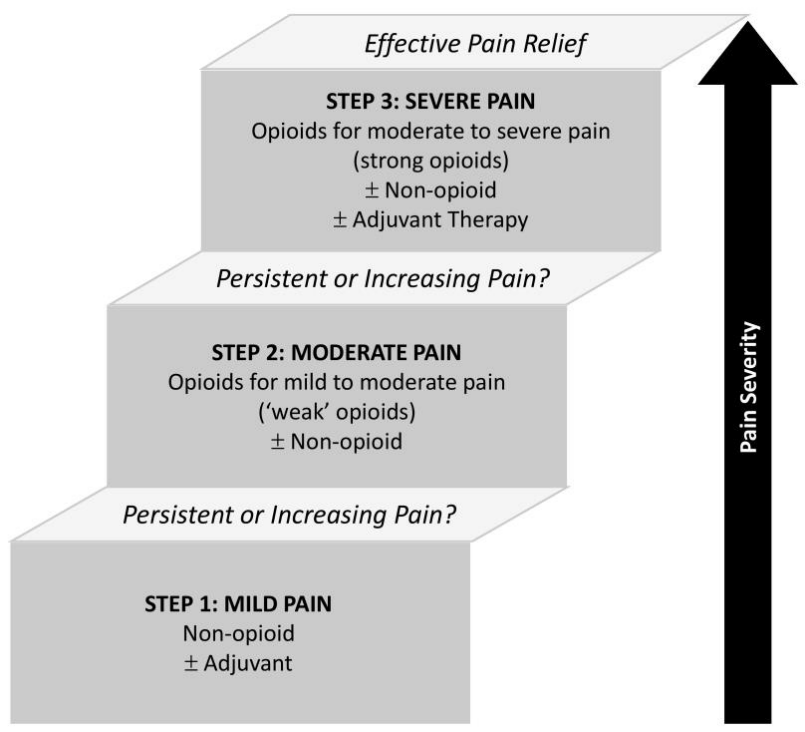
WHO Three-step Analgesic Ladder Adapted from the WHO Pain Relief Ladder.

A principal concept of the WHO guideline, the “three steps analgesic ladder,” refers to the pharmacological management of cancer-related pain (Figure 1). It emphasizes matching the strength of the analgesic drug to the intensity of the reported pain, so that non-opioid analgesics are administered for mild pain intensity, “weak” opioids for moderate pain, and “strong” opioids for severe pain. Non-opioid analgesics (simple analgesics) and non-steroidal anti-inflammatory drugs or adjuvant drugs (i.e. antidepressants or anticonvulsants) can be added at all steps according to the pain etiology. If pain is uncontrolled, the guideline recommends increasing opioid dose or rotating to a different drug of similar strength, adding a rescue medication or a non-opioid drug, or moving to the next step on the analgesic ladder. This simple approach has been proven effective in several confirmatory trials and has been recommended for use by all physicians caring for cancer patients.^6^,^7^ If pain is not improved within a short time, or if patients are experiencing intolerable side-effects of analgesia, they should be referred for more specialist advice and treatment in oncology, palliative care, or pain services.^8^

Regardless of the growing understanding of cancer pain mechanisms and treatment, and despite increasing efforts to disseminate the WHO guideline worldwide, only little progress has been made during the past three decades in terms of reducing the prevalence and severity of cancer-related pain.^1^ Many patients with advanced cancer continue to suffer moderate to severe pain and fail to receive analgesic drugs compatible with their pain intensities.^9^,^10^

International as well as several national guidelines on cancer-related pain have been published and disseminated in Israel.^7^,^11^,^12^ All recommended drug classes for cancer-related pain treatment are readily available and can be prescribed by all physicians in the country, regardless of their specialty. However, pain clinics in Israel, like in many other countries, have long waiting lists (many months)^13^ but nevertheless do tend to prioritize patients with difficult-to-treat cancer-related pain. Based on these facts, the present study’s hypothesis was that an adequate utilization of the WHO guideline is made by non-pain specialists prior to referring their patients with cancer-related pain to pain clinics (and therefore, pain specialists’ recommendations will mostly consist of interventions beyond the analgesic ladder). The present study aimed to test this hypothesis among physicians who referred cancer patients to a tertiary pain center in northern Israel.

## METHODS

The study was approved by the Ethics Committee at Rambam Health Care Campus in Haifa, Israel (approval number 0287-13).

### Patients

Both hospitalized patients and out-patients with cancer who were referred to a pain specialist for cancer-related pain management were recruited for the study. Inclusion criteria for the study consisted of being aged 18 and above; having a referral for cancer-related pain or cancer treatment-related pain; having the ability to understand the study aims; and giving written informed consent to participate in the study. Patients were excluded if their pain diagnosis was not related to cancer or cancer treatment, as determined by a pain specialist.

### Data Collection

Patients were asked to complete a questionnaire upon arrival to their consultation with a pain specialist at the Institute of Pain Medicine at Rambam Health Care Campus. The questionnaire consisted of questions regarding their demographic data; medical and cancer history; intake of pain medications during the previous 24 hours; medication-induced adverse effects; minimal, mean, and maximal pain intensity at rest and movement during the previous 24 hours (on a 0–10 numerical pain scale [NPS], where “0” is no pain and “10” is the worst pain imaginable); and the number of pain exacerbation episodes during the previous 24 hours. Data were also obtained on the recommended treatments noted in their physicians’ referral letters and in the medical records of hospitalized patients. Study participants also completed the short-form McGill Pain Questionnaire (SF-MPQ)^14^ and the Short Form Health Survey SF-12 (SF-12).^15^ When required, patients were given guidance and help with the questionnaires by an experienced nurse who was also responsible for the data collection (T.A.).

Pain specialists were informed that their patients’ data would be collected for the study. However, to avoid treatment selection bias, the specialists were not informed of the study’s aim.

### Data Analysis

The normality of data distribution of each variable was examined with the Shapiro–Wilk normality test. Data are presented as median (range) and/or mean± standard deviation (SD) for demographic variables, cancer parameters, pain intensities, SF-MPQ and SF-12 scores, and adverse effects.

The pain specialists’ consultations were retrospectively analyzed and categorized as “unjustified” or “justified” based on their treatment recommendation. Referrals to pain specialists were categorized “unjustified” if the specialists’ treatment recommendations remained within the WHO ladder framework and included one or more of the following four recommendations: (1) changing drug dose or rotating to an alternative drug within the same analgesic ladder step; (2) adding a rescue medication; (3) moving to the next step of the WHO analgesic ladder; or (4) adding an adjuvant treatment. Referrals were categorized “justified” when specialists’ recommendations fell outside of the WHO ladder in the following cases: (1) an invasive procedure; (2) administration of a systemic drug other than an opioid (including intravenous ketamine, lidocaine, bisphosphonates, etc.); (3) adding medical cannabis, which can only be carried out in Israel by a pain or palliative care specialist or by an oncologist; or (4) referral to radiation therapy, radio-isotope therapy, etc.

## RESULTS

### Patients

Complete data were available for 73 patients (44 women, 29 men). Their median age was 55 years (range, 25–85; mean±SD, 57±13).

### Cancer

Cancer type was heterogeneous, with breast cancer being the most common type (18%), followed by lung (14%) and colon (11%) cancer. Additional cancer types included pancreatic cancer, female reproductive cancers, and others (Table 1).

**Table 1:** Cancer Types in the Patient Cohort.

Cancer Type	Number of Patients (%)
Breast	13 (18)
Lung	10 (14)
Colo-rectal	9 (12)
Cervical	7 (9)
Ovarian	6 (8)
Pancreatic	6 (8)
Bladder	4 (5)
Other^*^	20 (25)

* Prostate, melanoma, lymphoma, mesothelioma, neuroblastoma, liver, myeloma, chondroma, thyroid, and kidney (1%–3% each).

Fifty-two patients (68%) had metastatic spread of their cancer to the following body regions: bones (26%), lungs (13%), liver (12%), other sites in the abdominal cavity (12%), lymph nodes (8%), and additional sites (10%). Notably, some patients (13%) had more than one metastatic site. The median cancer duration was 24 months (range, 1.5–276; mean±SD, 43±55).

### Pain

**Figure 2:**
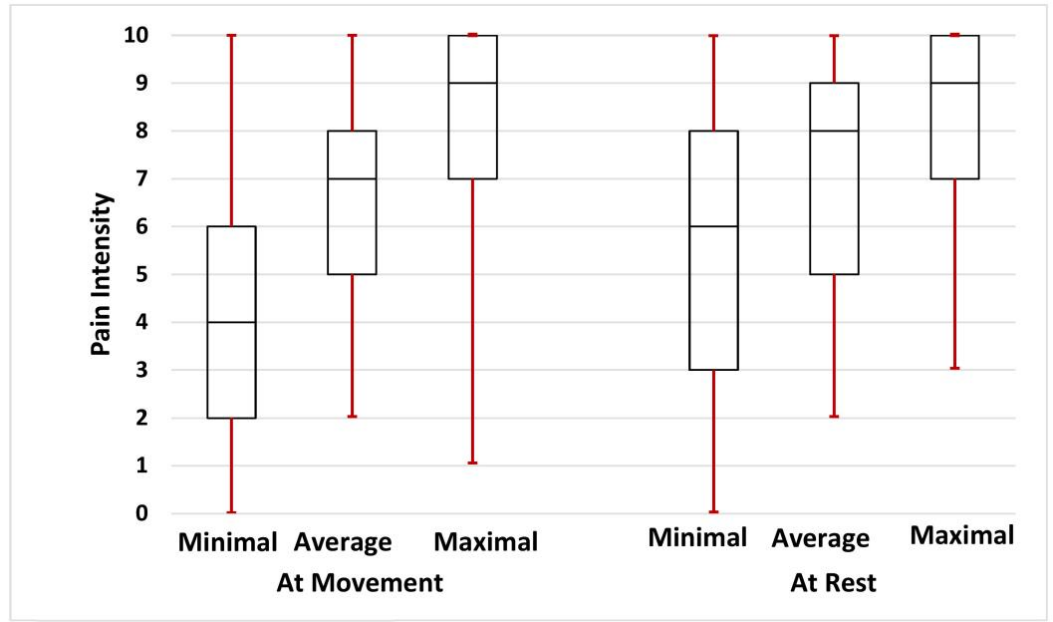
Minimal, Mean, and Maximal Pain Intensities at Rest and Movement. The minimal, average, and maximal resting and movement pain intensities are presented as median, quartiles (box), and range.

Pain duration was 6 months (range, 1–192; mean± SD, 15±27). Pain intensity at rest (NPS) was 7 (range, 2–10; mean±SD, 6.4±2.3) and at movement 8 (range, 3–10; mean±SD, 7.2±2.1). Figure 2 summarizes the minimal, average, and maximal resting and movement pain intensities. Importantly, at the time of referral 65 (85%) patients reported moderate to severe average pain intensity at rest, and 73 patients (96%) at movement.

Fifty-one patients (72%) experienced pain exacerbation episodes. The number of episodes was 2 (range, 1–30; mean±SD, 3.5±5.5). Although a majority of patients had fewer than 5 such episodes, four patients had 20, and one reported 30 pain exacerbation episodes.

The most frequent pain location involved the extremities in 36 patients (47%) and included focal extremity pain, pain radiation from another site, or in the form of distal neuropathy. The second most common sites involved the chest and the abdomen in 32 (42%), followed by upper and lower back in 30 (39%), pelvis in 19 (25%), and head and neck in 8 (10%). Most patients had more than one painful site.

Pain type was specified by a pain specialist as nociceptive in 52 patients (71%), neuropathic in 20 patients (27%), and visceral in 18 (25%). Hence, about one-quarter of the patients had a mixed type of pain. Pain was attributed to the cancer itself in 53 patients (73%) and to cancer treatment in 20 patients (27%).

### MPQ and SF-12 Questionnaires

Seventy-one patients (97%) completed the SF-MPQ, and all patients completed the SF-12 questionnaire. The median MPQ score was 25 (range, 3–45; mean±SD, 24±10). The median SF-12 physical component score was 23 (range, 17–25; mean±SD, 24±9), and the median mental component score was 35 (range, 22–36; mean±SD, 36±9).

### Treatment

At the referral evaluation, 56 patients (77%) were treated with opioids. Strong opioids alone were used by 51 patients, weak opioids were used by two, and a mixture of a strong and a weak opioid by three patients.

Of those using opioids, long-acting oral or transdermal preparations were used by 43 patients (77%), immediate-release opioids by 41 (73%), and rapid-onset opioids (ROOs) by two patients (3%) only. An additional 13 patients were treated by parenteral opioids, typically intravenous morphine infusions with or without rescue boluses. Non-steroidal anti-inflammatory drugs (NSAIDs) or cox-2 inhibitors (Coxibs) were taken by 12 patients (16%), simple analgesics by 32 (44%), and adjuvant drugs by 20 (27%). Herbal cannabis was used by five patients (7%). Almost all patients (88%) took the analgesic medications as recommended by their referring physician. In contrast, four patients took analgesics not prescribed by their caregiver, on their own initiative. An additional five patients were only partially compliant with their physician’s recommendations and avoided using specific analgesics or took them at a lower dosage than was recommended.

### Adverse Effects

The most frequently reported adverse effects in our study are summarized in Table 2.

**Table 2:** Frequencies and Severity of Adverse Effects.

Adverse Event	Mild	Moderate	Severe	Total
Dry Mouth	12 (16)	14 (19)	32 (44)	58 (79)
Tiredness	9 (12)	26 (36)	19 (26)	54 (74)
Mood Changes	10 (13)	19 (26)	11 (15)	40 (54)
Nausea	14 (19)	9 (12)	8 (11)	31 (42)
Constipation	4 (5)	7 (10)	18 (25)	29 (40)
Dizziness	7 (10)	12 (16)	8 (11)	27 (37)
Confusion	7 (10)	11 (15)	6 (8)	24 (33)
Sweating	4 (5)	14 (19)	6 (8)	24 (33)
Itching	5 (7)	10 (14)	4 (5)	19 (26)
Vomiting	3 (4)	4 (5)	4 (5)	11 (15)

Data presented as numbers of patients (%) reporting each adverse event.

### WHO Analgesic Categories

According to the administered medications, 69 patients (94%) were retrospectively categorized into one of the three WHO analgesic ladder steps. Three patients received only herbal cannabis, and one used only adjuvant drugs and therefore did not match any of the three formal steps. Fifty-four of the patients (74%) were compatible with step 3 of the ladder; three patients (4%) with step 2; and 12 patients (16%) with step 1. However, the mean pain intensity at rest and movement of the patients in all three WHO categories was 6 or above. This is also true for the four patients who received only adjuvant drugs or medical cannabis (Table 3).

**Table 3:** Baseline Pain Intensities at Rest and Movement According to the WHO Analgesic Treatment Step.

WHO Analgesic Step Used^*^	Pain at Rest	Pain at Movement
**Step 1 (*n*=12)**	7.3±2.6	7.1±2.4
	8 (2–10)	8 (3–10)

**Step 2 (*n*=3)**	6.3±1.1	5±0
	7 (5–7)	5 (5–5)

**Step 3 (*n*=54)**	6.2±2.3	7.3±2
	7 (2–10)	8 (3–10)

**Medical Cannabis Only (*n*=3)**	6.6±1.5	7.3±1.5
	7 (5–8)	7 (7–9)

**Adjuvant Drugs Only (*n*=1)**	7	8

Data presented as mean±SD, median (range).

* WHO step as determined by the type of analgesic medication consumed.

Spearman’s coefficient correlations were used to test relationships between the WHO analgesic step medications used and the mean pain intensities. No significant correlations could be demonstrated either at rest (*r*=0.31, *P*=0.807) or at movement (*r*=0.33, *P*=0.812).

As mentioned earlier, 20 patients (27%) were diagnosed with neuropathic pain by a pain specialist. Of them, only 10 patients received adjuvant treatments in an attempt to treat the neuropathic pain.

### Source of Referral

All patients were referred to pain specialist consultation by their physicians: 49 (67%) by hospital physicians; 24 (33%) by community physicians; 17 (23%) by internists; 15 (21%) by family practitioners; 10 (14%) by gynecologists; nine (12%) by oncologists; and six (8%) by surgeons. The remaining 16 (22%) patients were referred by other physicians from various medical fields.

### Treatment Recommendations from Pain Specialists

For 62 (85%) patients, recommendations remained within the WHO ladder as follows: 24 patients (33% of all patients in the survey) were advised to change opioid analgesic dose; in four patients (5%) an adjuvant medication was added; 10 patients (14%) were advanced to the next WHO analgesic step; and eight patients (11%) were given a rescue medication. Sixteen patients (22%) required two simultaneous changes within the ladder (i.e. both an increasing opioid dose and the addition of a rescue medication).

The remaining 11 patients (15%) required an intervention not included in the WHO ladder: in nine patients (11%) medical cannabis was prescribed either alone (*n*=5) or in combination with other changes in the medical regimen within the WHO ladder. One patient was advised to undergo a nerve block, and another received a prescription for methylphenidate to manage opioid-induced excessive daytime sleepiness. As mentioned earlier, a recommendation for cannabis use in Israel can only be completed by a specialist in pain, palliative medicine, or oncology. Since none of the patients for whom medical cannabis was recommended were referred by an oncologist, their referrals were considered “justified.” Similarly, methylphenidate is not registered in Israel for reducing opioid-induced sedation, and its administration requires a special recommendation from a pain specialist.

### Justification of Referral to Pain Specialist

A referral to a pain specialist was categorized “justified” when the specialist’s recommendation was outside of the WHO analgesic ladder. Only 11 patients (15%) were advised to receive a treatment that is not part of the WHO analgesic ladder. Most of these patients (six patients) were referred from a community clinic by their family practitioner. The rest were referred from the hospital by an internist (three patients), an oncologist (one patient), or an occupational physician (one patient). Conversely, the majority of referrals (85%) were categorized as “unjustified.”

## DISCUSSION

Although cancer pain represents a complex medical condition, the WHO analgesic ladder has a simple and logical structure, allowing widespread implementation by all physicians caring for cancer patients. Validation studies have shown that up to 73% of cancer-related pain can be reduced when the WHO guideline is properly implemented.^6^ However, in practice, sufficient pain control is achieved in only 50% of the patients.^5^ The question of why this happens remains open.

Congruent with the literature,^1^ the patients referred to our pain center with advanced cancer and a high percentage of metastases reported severe resting and movement pain on average and frequent episodes of pain exacerbation during the day. In addition, they exhibited impairments in both the mental and physical components of quality of life, as indicated by their responses on the SF-12 questionnaire.

The main finding in the present study is that, contrary to our hypothesis, 85% of patients were advised by a pain specialist a treatment within the framework of the WHO three-step ladder. This implies that the pharmacological management of cancer-related pain provided by the WHO guideline was not reasonably utilized by non-pain specialists who care for such patients. A second important finding is that patients exhibited high compliance with the analgesic treatment prescribed by their referring physicians. Close to 90% of the patients reported analgesic consumption that was consistent with the analgesic regimen documented in their referral letter or in their hospital medical record. This is despite the high percentage of reported adverse effects by the patients in our study. These two findings imply that patients’ concerns about treatment side-effects are not the main reason for the observed suboptimal treatment in the patients. Rather, the main problem seems to be an incomplete implementation of the WHO guideline by non-pain specialist physicians. As shown in the literature,^16^ up to 31% of physicians tend to delay the use of strong opioids until a patient is terminal or suffering from intolerable pain. In a number of studies only approximately half of the primary physicians chose to treat severe cancer pain with strong opioids.^17^–^19^ This may be due to physicians’ concerns such as adverse effects of opioids, fear of potential addiction, or patients becoming tolerant to opioids.^20^

Several additional factors point to significant gaps in the implementation of the WHO guideline for the management of cancer-related pain. First, 16 patients (22%) did not receive opioids although their reported mean pain was moderate to severe (with an NPS of 6 or greater). These patients were treated by either simple analgesics, an adjuvant drug, or medical cannabis. Notably, in recent years medical cannabis has emerged as a new player in the cancer-related pain treatment armamentarium and can be prescribed for this indication in at least some countries, although evidence for its effectiveness is still anecdotal at best.^21^,^22^ This suggests that at least some physicians consider simple analgesics or medical cannabis a substitute for opioids for moderate to severe cancer pain. Second, of the 20 patients diagnosed by a pain specialist as having neuropathic pain, only 10 received an adjuvant drug, although adjuvant treatments are recommended for this type of pain.^23^,^24^ Neuropathic pain is a complex problem since there is not only a lack of adherence to the WHO treatment guideline but also difficulties with diagnosing it.^25^

It is important to refer patients to pain specialist consultations if pain is not improving within a short time or if patients are experiencing intolerable side-effects of analgesia. However, given the poor availability of pain clinics, attempts to optimize analgesic treatment based on the WHO guideline are expected to be made prior to such referrals. Although all referred patients in the present study were treated prior to their referral, such treatment was not successful, and 85% of the patients were provided treatment by their pain specialist that is within the WHO ladder framework. We have not investigated the reasons for this poor adherence to the WHO guideline. Several providers’ barriers have been identified in the past, such as poor pain assessment, insufficient knowledge about pain treatments, and prioritizing cancer treatment over pain treatment.^26^ In a US national survey, Breuer et al. reported that oncologists rated their ability to manage cancer pain as high (i.e. a median of 7 on a 0–10 scale), while acknowledging their poor pain management training. Though most oncologists agreed with basic pain treatment principles, for example the importance of opioid therapy, the majority displayed knowledge deficits about such therapy in the context of cancer pain.^27^ Physicians may base their clinical decisions primarily on their personal experience, even when clinical guidelines may advise different treatment strategies. Breuer et al. suggest that better practices in pain management are necessary, and this need cannot be met through referrals to pain specialists.^27^ Furthermore, in a large cross-sectional survey, 50% of cancer patients believed that their quality of life was not considered a priority in their overall care by their healthcare physician.^4^ Many patients feel that their physician prioritizes cancer treatment over pain treatment. This is reflected in poor pain assessments and limited time dedicated to pain in consultations. Moreover, the decision-making of healthcare providers is influenced by concerns such as regulations, adverse effects of analgesics (primarily opioids), fear of potential addiction, or patients becoming tolerant to the analgesic drug.^20^

However, patient-related barriers such as fears or unwillingness to take pain medication^28^ and healthcare systems’ restrictions such as over-regulation on narcotics use have also been reported in the literature.^29^ As mentioned earlier, we do not think that these barriers apply to our patients. Most patients adhered to their physician’s instructions, and no restrictions on opioid administration, dose limitation, or accessibility exist in Israel.

Our findings and those of others^16^ point to the current lack of any strategy to systematize guideline implementation as the core of the problem. It is still likely that some oncologists prioritize cancer treatments over cancer-related pain management, leaving pain treatment and management to pain specialists. However, a systematized implementation program may be effective in changing healthcare professionals’ behavior, which is so necessary to improve cancer-pain management.

One possible limitation of our study is the lack of a follow-up session for evaluating the outcome of the pain specialists’ recommendations. Seemingly, this could have resulted in a more comprehensive clinical picture. However, this was clearly beyond the scope of this study, which focused primarily on referrals to pain clinic consultations rather than on the outcome of these consultations.

## CONCLUSION

The pharmacological management of cancer-related pain provided by the WHO guideline was not reasonably utilized by non-pain specialists in our study. This is despite the high compliance of patients with the analgesic treatment prescribed by their referring physicians. Future studies should aim at a better understanding of cancer-related pain management barriers and improving pain management guideline adherence.

## Abbreviations

NPS: numerical pain scale
SD: standard deviation
SF-12: Short Form Health Survey SF-12
SF-MPQ: short-form McGill Pain Questionnaire
WHO: World Health Organization.
